# A Cutaneous Vascular Neoplasm With an EWSR1‐NFATC2 Translocation—Contributing to the Spectrum of Vascular Lesions Characterized by NFATC‐Related Fusions

**DOI:** 10.1111/cup.14800

**Published:** 2025-03-04

**Authors:** B. Schurink, A. M. van Huizen, C. D. Savci‐Heijink, E.‐J. Kooi

**Affiliations:** ^1^ Department of Pathology Amsterdam University Medical Centers the Netherlands; ^2^ Department of Dermatology Amsterdam University Medical Centers the Netherlands

**Keywords:** epithelioid vascular tumor, EWSR1—NFATC2, hemangioma, skin

## Abstract

Recently, a distinct subgroup of vascular neoplasms has been identified, characterized by *NFATC*‐related fusions. Although existing literature is limited, these lesions histologically show a variable appearance with a tendency for local recurrence but not distant spread. Therefore, they likely fall within the “benign” or at most in the “local aggressive”/“borderline” tumor category according to the International Society for the Study of Vascular Anomalies (ISSVA) classification scheme. Up to now, vascular tumors with *NFATC*‐related fusions have only been documented in bone and occasionally soft tissue. We present a case of a woman with a “difficult‐to‐diagnose” multifocal cutaneous vascular neoplasm showing an *EWSR1::NFATC2* translocation. To our knowledge, this is the first report on a vascular neoplasm with an *EWSR1::NFATC2* translocation occurring in the skin.

## 1 Introduction

Diagnosing vascular anomalies can be particularly challenging due to their broad clinical and histological spectrum, which often includes overlapping features and requires accurate clinical, radiological, and pathological correlation. Our understanding of these lesions is slowly improving, largely due to the elucidation of their underlying genetic alterations. A good example is the group of epithelioid vascular tumors characterized by an *NFATC*‐related fusion. Thus far, vascular tumors harboring an *NFATC1/C2* fusion have only been documented in bone and occasionally soft tissue [1, 2, 3, 4]. Here, we present a challenging case of a multifocal cutaneous vascular neoplasm, which ultimately revealed an *EWSR1::NFATC2* translocation. This is the first case reporting on a dermal vascular lesion characterized by an *NFATC*‐related fusion.

### 1.1 Case Report

**FIGURE 1:**
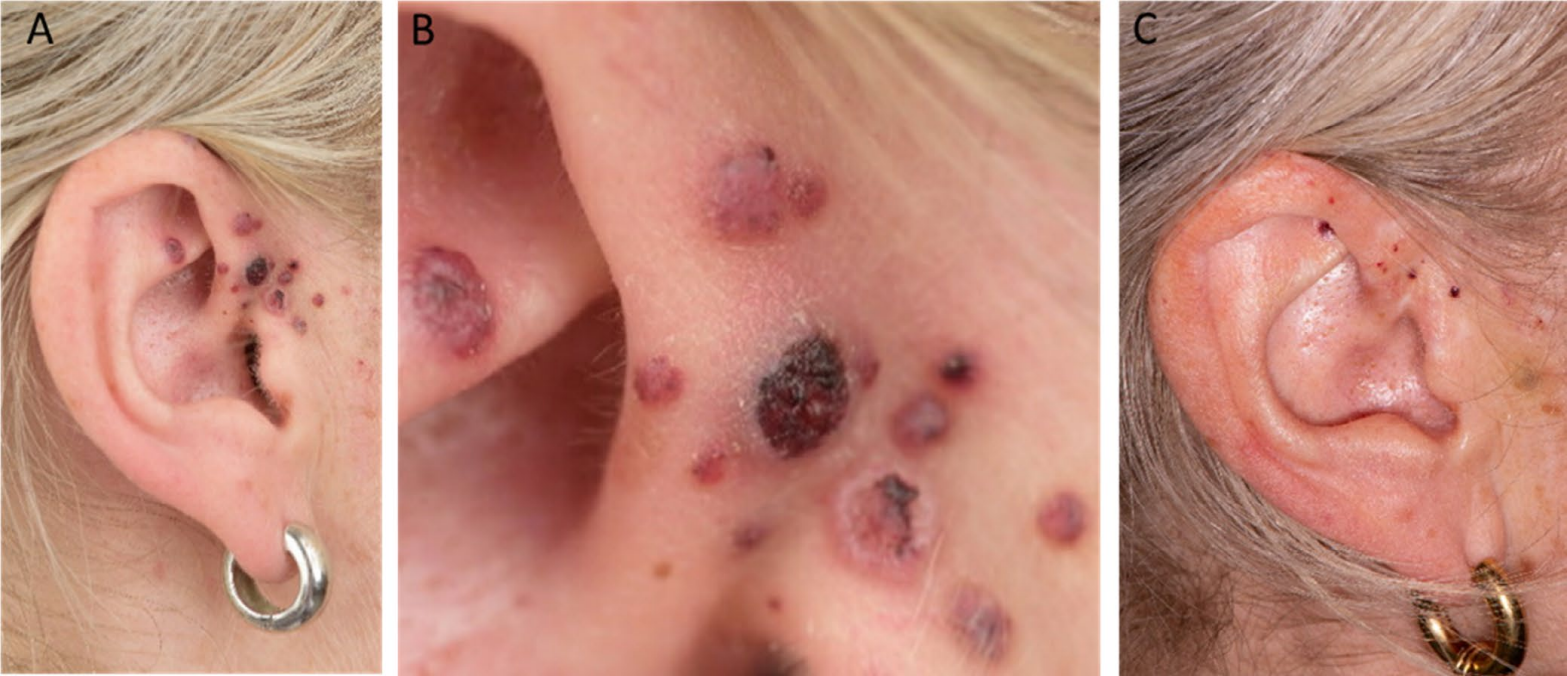
Clinical appearance of the lesions. (A, B) Initial presentation at our hospital several years ago, showing multiple hyperkeratotic hemorrhagic lesions distributed on and around the right ear. (C) Current appearance of the lesions after extensive laser treatment.

**FIGURE 2:**
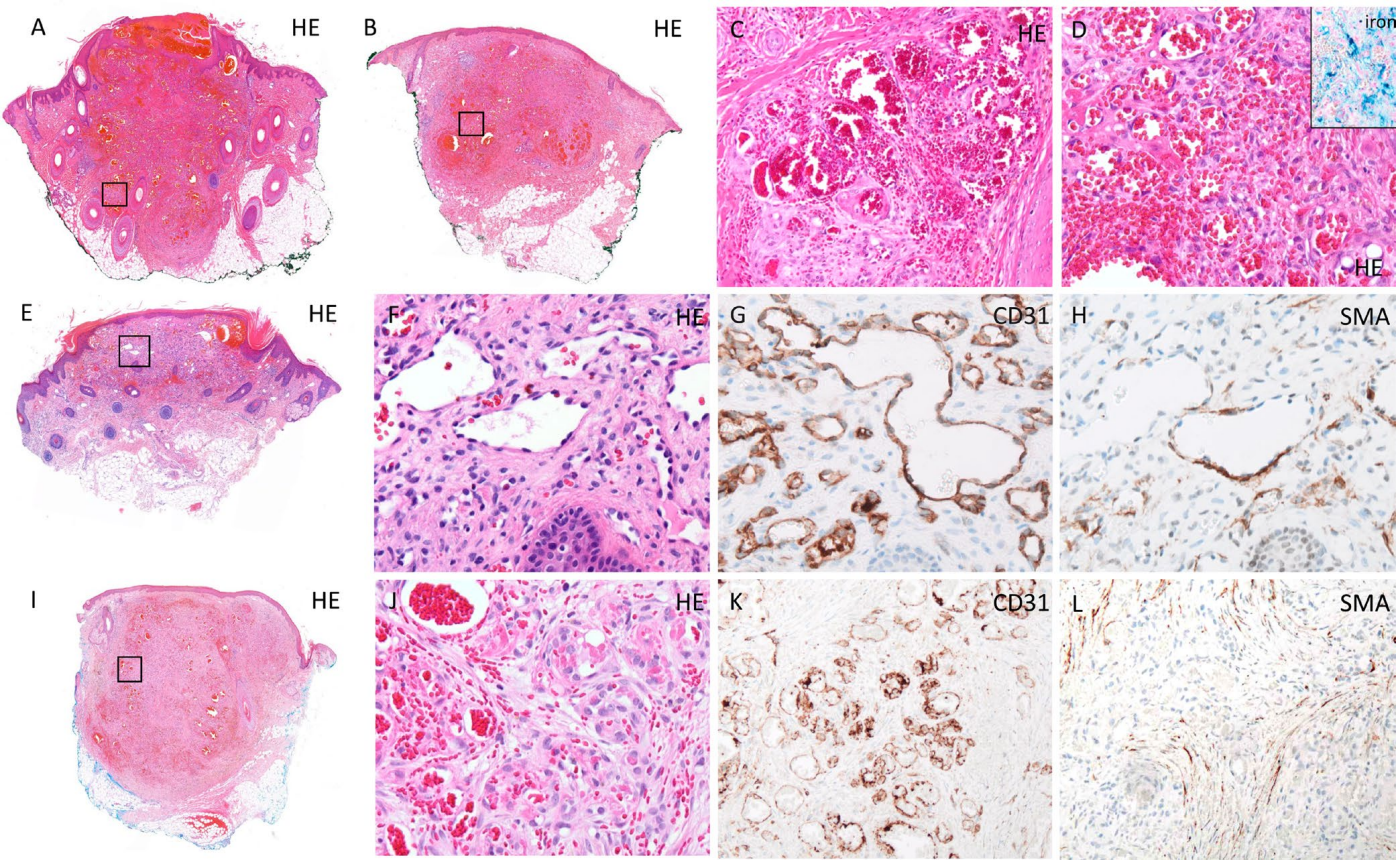
Histopathologic images of the lesions over time. (A–D) Two of the three initially excised (untreated) lesions showing the varying sizes on low‐power view (A, B; 6×, H&E). They displayed a vaguely lobular arrangement (C; 210×, H&E) consisting of capillary‐sized vessels with flat (C) to somewhat plump endothelium (D; 210×, H&E), extensive hemorrhage and significant iron deposition (D, inset, 100×, Perls' iron stain). (E–H) Diagnostic biopsy of a lesion after referral to our hospital reveals a similar relatively well‐circumscribed vasoformative lesion (E; 6×, H&E), sometimes somewhat resembling retiform‐like growth with plump/hobnailed endothelium (F; 200×, H&E), but lacking pleiomorphism, multilayering or brisk mitotic activity. The lesional vessels are lined with endothelial cells that were positive for CD31 (G; 200×, CD31) and negative for D2‐D40 (not shown) and in many areas, lack a pericytic layer as evidenced by smooth muscle actin (SMA) immunohistochemistry (H; 200×, SMA). (I–L) Example of one of the two diagnostic biopsies of recurrent lesions approximately 4 years after referral to our hospital. Low‐power image shows a fairly well‐circumscribed vasoformative lesion (I; 6×, H&E), which on high‐power view shows extensive hemorrhage and capillary‐sized vessels, and partly poorly formed vessels with epithelioid endothelial cells (J; 210×, H&E). The endothelial cells of the lesional vessels were positive for CD31 (K; 160×, CD31) but largely lacked a pericytic layer, with varying amounts of spindly myofibroblasts throughout the lesions (L; 160×, SMA).

Our patient, a 41‐year‐old woman with no significant medical history, was referred to our hospital because of recurring, multifocal hyperkeratotic erythematous papules on the right ear and temporal side of the head (Figure 1A,B). At the time of referral, these lesions had been present for over 2 years and had already been partially resected at another hospital (three lesions in total). As part of the referral process, the histology was re‐reviewed (Figure 2A–D). At our dermatology policlinic, a new biopsy was performed (Figure 2E–H). Combined with the clinical presentation, the lesions were initially classified as pyogenic granuloma with satellitosis [5]. However, the histological findings were considered nonspecific. Subsequently, therapy with timolol 0.5% XE gel was initiated, which had insufficient effect on the lesions. Consequently, the most prominent lesions were removed using pulsed dye and Nd:YAG lasers (Figure 1C). Unfortunately, despite laser application, the lesions recurred.

To achieve a more conclusive diagnosis, two additional biopsies were obtained (Figure 2I–L) approximately 5 years after referral to our hospital. Considering all lesions together (a total of six), histological examination revealed lesions of varying sizes (Figure 2A,B,E,I), characterized by relatively well‐demarcated borders and absence of infiltrative growth as well as a conspicuous inflammatory infiltrate. Moreover, the lesions showed remarkable hemorrhagic changes, with extensive iron deposition evident in all lesions (Figure 2D), along with occasional formation of microthrombi. The lesions displayed a varied morphological appearance. Parts of the lesion were reminiscent of a composite hemangioendothelioma, with some areas somewhat resembling retiform‐like vascular channels lined with plump hobnailed endothelium (Figure 2F); other areas resembled hemangioma‐like areas arranged in a vaguely lobular fashion with capillary‐sized vessels lined by flat endothelium (Figure 2C), while another component displayed a striking epithelioid morphology of the endothelial cells (Figure 2J). Notably, there was an absence of multilayering of endothelial cells, as well as any evident cytonuclear atypia or brisk mitotic activity. Additionally, notable “reactive changes” were observed, including epidermal hyperplasia and areas demonstrating fibroblast proliferation. Immunohistochemistry revealed a blood‐related angiomatous signature, as evidenced by positive staining for CD31 and ERG, while lymphatic differentiation was absent (negative for D2‐40; not shown). Interestingly, the majority of the lesional vessels exhibited a striking absence of a pericyte layer, as indicated by largely negative or discontinuous staining for smooth muscle actin (SMA; Figure 2H,L), a consistent finding across all sampled lesions. HHV8 was consistently negative, effectively ruling out Kaposi sarcoma. Synaptophysin, FOS(B), and pankeratin immunostainings were negative as well. Based on its complex morphology and immunohistochemical signature, classification of the vascular lesions remained challenging with a broad differential diagnosis including a variant hemangioma (e.g., epithelioid hemangioma or low‐grade/“intermediate” vascular tumor [like composite hemangioendothelioma]). Subsequent molecular analyses were performed, revealing no mutations through next‐generation sequencing. Archer FusionPlex PCR assay identified an *EWSR1::NFATC2* translocation (Exons 3–8 [NM_005243.4—NM_012340.5]).

To rule out metastatic disease, a whole body PET‐CT and MRI of the brain were performed. Apart from an incidental finding (a radiological lesion suggestive of an epidermoid cyst at the cerebellopontine angle), there was no evidence of metastatic disease. Despite a relatively limited follow‐up period of approximately 7–8 years since the initial occurrence of lesions, the course has been benign, only characterized by a tendency for local recurrence, but no evidence of distant metastases, consistent with existing literature. The patient will continue Nd:YAG laser treatment for the remaining lesions, and regular clinical follow‐up will be maintained.

## 2 Discussion

Thus far, vascular tumors harboring an *NFATC1/C2* fusion have only been documented in bone and occasionally soft tissue [1, 2, 3, 4]. With the description of this case, we aim to contribute to the limited literature available on vascular neoplasms with *NFATC*‐related fusions and raise awareness among clinicians and pathologists that these lesions may primarily manifest in the skin.

Based on H&E staining combined with immunohistochemistry, the lesion was deemed to be vasoformative, with a broad differential diagnosis due to its complex morphology. In the absence of overt malignant features—such as infiltrative growth, necrosis, brisk mitotic activity, or high‐grade cytonuclear atypia—an angiosarcoma was considered unlikely. The absence of myxohyaline stroma and distinctive vascular channel formation distinguished the lesion from a (classical) epithelioid hemangioendothelioma, although a variant with an alternative YAP1‐TFE3 fusion remained a possibility [6, 7]. The lesion's variegated morphology, including focal retiform‐like vascular channels, raised considerations of retiform hemangioendothelioma or composite hemangioendothelioma [8, 9]. However, these were deemed somewhat less likely due to the absence of classic histological features, no evidence of lymphatic differentiation, and negative synaptophysin, which is present in a subset of these lesions. The epithelioid phenotype observed in a proportion of endothelial cells suggested a potential diagnosis of epithelioid hemangioma. However, this was questioned due to the absence of an eosinophilic infiltrate, lack of FOS(B) staining, and lack of pericytes surrounding most vessels, which would be atypical for such a lesion.

Given the lesion's unusual morphology, its lack of alignment with known vascular skin lesions, the recurrent behavior, and the worrisome absence of pericytes surrounding most vessels, molecular analyses were conducted. These analyses identified an *EWSR1::NFATC2* translocation, and based on these findings, the lesion was classified as a vascular neoplasm with an *NFATC*‐related fusion [3]. Despite some worrisome histological features and a propensity for local recurrence, this molecularly distinct vascular neoplasm, as evidenced by its lack of distant spread, likely falls within the “benign” or at most in the “local aggressive”/“borderline” tumor category according to the International Society for the Study of Vascular Anomalies (ISSVA) classification scheme [10]. Acknowledging this likely under‐recognized distinct entity is important for prompt and accurate diagnosis, treatment, and follow‐up care, while also facilitating a better understanding of its complete clinical and histological spectrum and exact biological behavior.

## Author Contributions

All authors made substantial contributions to conception and design, to acquisition of data, or to analysis and interpretation of data; involved in drafting the manuscript or revising it critically for important intellectual content; given final approval of the version to be published. Each author should have participated sufficiently in the work to take public responsibility for appropriate portions of the content, and agreed to be accountable for all aspects of the work in ensuring that questions related to the accuracy or integrity of any part of the work are appropriately investigated and resolved.

## Ethics Statement

Informed consent for publication was obtained from the patient.

## Conflicts of Interest

The authors declare no conflicts of interest.

## Data Availability

Data sharing is not applicable to this article as no new data were created or analyzed in this study.
